# Serum tumor necrosis factor alpha increased during remission with Etanercept

**DOI:** 10.1186/1546-0096-9-S1-P108

**Published:** 2011-09-14

**Authors:** Mihaela Spirchez, Gabriel Samasca, Claudia Bolba, Nicolae Miu

**Affiliations:** 1 Department of Pediatrics, 2nd Pediatric Clinic, „Iuliu Haţieganu” University of Medicine and Pharmacy Cluj-Napoca, Romania; 2 Department of Immunology, „Iuliu Haţieganu” University of Medicine and Pharmacy Cluj-Napoca, Romania

## Background

Existing medical evidence regarding cytokine profile in both plasma and synovial fluid support significant changes in Juvenile Idiopathic Arthritis (JIA) patients.

## Aim

To evaluate the possible role of TNF-α in monitoring disease activity in JIA.

## Methods

In a 2-year prospective study, TNF-α levels were measured using ELISA in 63 serum samples and 4 synovial fluid (SF) samples, for 40 JIA patients. The control population consisted of 18 healthy children. The data were correlated with disease activity and severity (quantified with JADAS27 score) and also with several biomarkers of inflammation.

## Results

TNF-α was measurable in 39 serum samples and in all SF samples. Only 19% of serum samples had greater TNF-α levels than controls. The most elevated levels of serum TNF-α were found in patients during clinical remission with Etanercept. In one patient with seropositive polyarticular disease, Etanercept administration for 6 months resulted in important elevation of serum TNF-α, regardless of clinical improvement. SF levels were significantly higher than simultaneously serum levels (p=0.02), with no significant differences among oligoarticular and polyarticular forms of JIA. We found no correlation of serum TNF-α values with disease activity and severity. In our patients the circulating TNF-α values were significantly correlated only with hemoglobin levels (r=-0.30; p=0.04) in the oligoarticular group and with serum Immunoglobulin G (r=0.70; p<0.01) in the systemic group.

## Conclusions

We found no correlation of serum TNF-α values with disease activity and severity. Upon treatment with Etanercept, although many JIA patients reached remission on medication, they developed increased circulating TNF-α levels.

